# Management of Acute Ischemic Stroke Following Transcatheter Aortic Valve Implantation: A Systematic Review and Multidisciplinary Treatment Recommendations

**DOI:** 10.3390/jcm13237437

**Published:** 2024-12-06

**Authors:** Matthew Hammond-Haley, Ahmad Almohtadi, Ahmed R Gonnah, Oishik Raha, Arif Khokhar, Adam Hartley, Saud Khawaja, Nearchos Hadjiloizou, Neil Ruparelia, Ghada Mikhail, Iqbal Malik, Soma Banerjee, Joseph Kwan

**Affiliations:** 1 National Heart and Lung Institute, Hammersmith Hospital, Imperial College London, London SW7 2BX, UK; 2 Oxford University Hospitals NHS Trust, Oxford OX3 9DU, UK; 3 Department of Brain Sciences, Imperial College London, London SW7 2BX, UK

**Keywords:** TAVI, TAVR, stroke, acute ischemic stroke, thrombolysis, mechanical thrombectomy

## Abstract

**Background/Objectives**: Acute ischemic stroke is an uncommon but potentially devastating complication of Transcatheter Aortic Valve Implantation (TAVI). Despite improvements in device technology and procedural techniques, stroke rates have remained stable, with cerebral embolic protection devices demonstrating only limited efficacy to date. Therefore, the management of acute ischemic stroke complicating TAVI (AISCT) remains a key priority. We conducted a systematic review of the management of AISCT and provided multidisciplinary consensus recommendations for optimal management. **Methods**: PubMed, Google Scholar, and Cochrane databases were searched from inception to October 2023. All the original studies focusing on the treatment of AISCT were included. Non-English language studies, review articles, and studies in pediatric populations were excluded. Consensus recommendations were made by a working group comprising experts in stroke medicine and structural interventional cardiology. **Results**: A total of 18 studies met the inclusion criteria, including 14 case reports/series and 4 observational studies. No clinical trials were identified. The included case reports and series suggest that tissue-type plasminogen activator (tPA) and mechanical thrombectomy (MT) might be effective strategies for managing AISCT. However, significant bleeding complications were reported in two out of the four patients receiving tPA. Four observational studies also suggest an association between tPA and/or MT and improved functional outcomes and survival compared to conservative management. Higher bleeding rates were reported following tPA. Observational data suggest that there is currently little real-world utilization of either reperfusion strategy. **Conclusions**: There is an absence of high-quality randomized data to guide clinical decision making in this important area. Observational data suggest reperfusion strategies are associated with improved clinical outcomes once important confounders such as stroke severity have been accounted for. While MT can be recommended as the standard of care in appropriately selected patients, significantly increased rates of bleeding with tPA following large-bore arterial access raise important safety concerns. We present simple clinical guidance for AISCT based on the limited available data. Close multidisciplinary work and patient-specific consideration of ischemic and bleeding risk is essential.

## 1. Introduction

Transcatheter Aortic Valve Implantation (TAVI) has revolutionized the management of symptomatic severe aortic stenosis over the last two decades, and is now the standard of care with a class IA recommendation in patients over the age of 75 years regardless of surgical risk [1,2]. Recent trials have demonstrated favorable results even in low surgical risk populations, maintained at four and five years for the most widely used self-expandable and balloon-expandable TAVI platforms, respectively [3,4]. The net result, in the context of an aging population, has been a rapid uptake of TAVI worldwide with TAVI now outnumbering surgical aortic valve replacement (SAVR) in many countries, with procedural numbers forecast to continue to rise over the coming decades [5].

Stroke is an uncommon but potentially devastating complication of TAVI. Major disabling acute ischemic stroke complicating TAVI (AISCT) is defined by a focal or global neurological deficit with a modified Rankin scale (mRs) of 2 or higher within 90 days [6]. Rates of major disabling stroke were comparable to SAVR in the early high-surgical-risk trials and significantly lower than SAVR in lower-risk patient cohorts [3,4]. Despite improvements in AISCT risk prediciton, with numerous patient-related (e.g., age, previous stroke, calcium score, and atheromatous aorta) and procedural risk factors (balloon- vs. self-expandable valves, post dilatation) identified [7], the overall incidence of AISCT remains approximately 2–3% in real-world contemporary registry data [3]. Outcomes after AISCT are poor, with a greater than five-fold increase in mortality at one year and significantly impaired quality of life [8,9,10]. As TAVI numbers expand, AISCT will account for growing morbidity and mortality and place a substantial strain on healthcare resources. As such, evidence-based approaches to the prevention and management of TAVI-related strokes are urgently needed.

It is important to consider the potential pathophysiological mechanisms in this patient cohort, as usual dogmas and established practises in stroke medicine may not be directly applicable. The timing of stroke post-TAVI gives some indication of the likely etiology. Approximately 50% of AISCT occurs within the first 24 h following TAVI, in which case procedural factors such as embolization (valve material, atheromatous material or calcific debris, and thrombus or air) and/or hypoperfusion are most likely. Histological studies of debris extracted from cerebral embolic protection (CEP) devices demonstrate the presence of arterial wall tissue, native valve tissue, calcifications, and percutaneous device foreign material [11,12,13,14]. Transcranial Doppler studies demonstrate peaks during valve positioning and implantation, suggesting the calcified stenotic native aortic valve is the likely primary origin of microembolization [15]. Importantly, many of these materials are unlikely to be disrupted by conventional pharmacological thrombolysis [16]. Stroke risk can, however, be elevated for several months following TAVI [17]. Subacute strokes (24 h to 30 days) are more likely related to patient factors, including atrial arrhythmias and baseline atherosclerotic risk factors pre-disposing to stroke [8].

While stroke prevention during TAVI has been a focus of the Heart Team research community, cerebral embolic protection (CEP) devices have only demonstrated limited efficacy [17,18,19], with further randomized control trials (RCTs) ongoing [20]. The treatment of AISCT should, therefore, remain a priority; however, there are no specific guidelines in this area. Current emergency management options for acute ischemic stroke include single or dual antiplatelet therapy, intravenous thrombolysis, and mechanical thrombectomy (MT) [21]. How these treatment options should be employed in the setting of AISCT remains unclear. This study aims to systematically evaluate the literature on AISCT treatment and provide multidisciplinary consensus recommendations for early identification and initial management.

## 2. Methods 

### 2.1. Search Strategy

We conducted a systematic search of PubMed, Cochrane, EMBASE, and Google Scholar from inception up to October 2023 in accordance with the PRISMA guidelines [22]. The following Boolean query was used: “Stoke” AND (“TAVI” OR “TAVR” OR “transcatheter aortic valve implantation” OR “transcatheter aortic valve replacement”) AND (“aspirin” OR “thrombolysis” OR “tPA” OR “alteplase” OR “Tenecteplase” OR “thrombectomy”) across all the databases.

### 2.2. Study Selection

The titles and abstracts identified by the search were independently extracted and reviewed by two investigators (M.H.H and A.A). The reference lists of the included studies, as well as pertinent reviews, were also screened to identify any additional relevant studies. Data were independently collected pertaining to study design, patient characteristics, stroke severity, aortic stenosis severity, stroke diagnosis, stroke management, outcomes, and follow-up. Any discrepancies in the included studies or data collected were resolved by a third independent reviewer (A.R.G).

### 2.3. Eligibility Criteria

All the original studies focusing on the treatment of acute ischemic strokes following TAVI were included. Review articles, editorial comments, studies focusing on the prevention of stroke in patients undergoing TAVI, studies in pediatric population (<18-year-old), and non-English language publications were excluded.

### 2.4. Working Group

A working group comprising experts in structural interventional cardiology and stroke medicine at Imperial College Healthcare NHS Trust was formed. Tertiary hyperacute stroke services at Imperial College Healthcare NHS Trust include a 24 h, 7-day-a-week MT service that receives referrals from surrounding areas in North West London. Approximately 300 MTs for stroke are performed each year. Tertiary cardiology services are based at a separate hospital site, five kilometers apart, where approximately 300–350 TAVI procedures are performed each year. Members of the working group between sites collaboratively assessed the available evidence and provided their consensus and recommendations on the hyperacute management of AISCT.

## 3. Results

**Figure 1:**
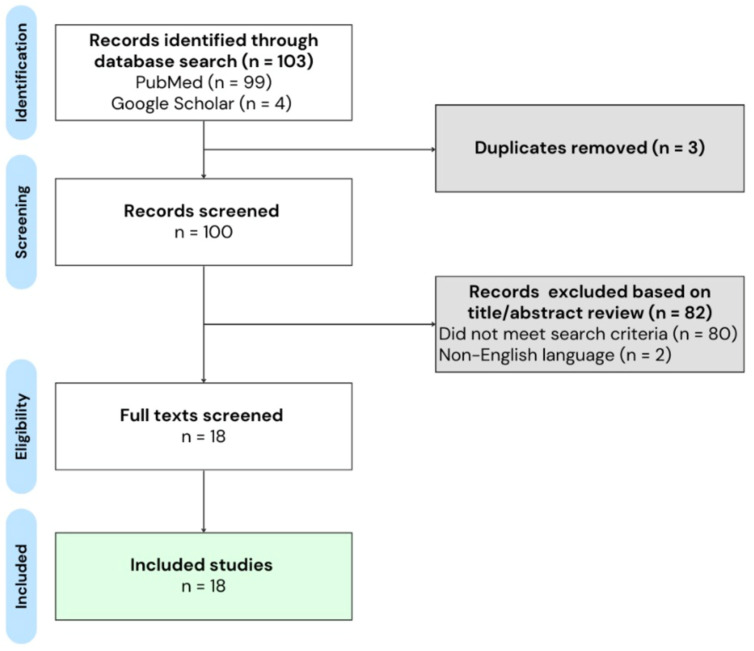
PRISMA flow diagram.

A total of 103 studies were identified from the initial search strategy. After removing three duplicates, 18 studies including 1617 patients met the inclusion criteria (Figure 1). In total, 14 (77.8%) of the studies included were case reports of single patients, and 2 (11.1%) were case series of two patients. Four observational studies were also included. One study (5.6%) was a small retrospective observational study of 22 patients, 8 of whom received tPA for AISCT while 14 received conservative management. Three studies (16.7%) were large observational registry studies, including a total of 1576 patients. No randomized clinical trials were identified.

### 3.1. Case Reports and Case Series

Baseline patient characteristics and a summary of the findings from te case reports and series are provided in Table 1. The patients were elderly with a mean age of 82 ± 3.486 years, with a moderate burden of cardiovascular co-morbidity. Stroke syndromes were large, with a baseline NIHSS of 8.5 ± 3.3 and 18.3 ± 2.8 in the tPA and/or MT groups, respectively.

Four patients received tPA, resulting in successful outcomes with documented reductions in NIHSS scores: one patient was asymptomatic at follow-up, another reduced NIHSS from 10 to 2, another from 17 to 2, and the last from 5 to 3 [23,24,25,26]. Complications included significant bleeding from the femoral access site and bilateral groin hematomas [25,26]. Mechanical thrombectomy was performed on 13 patients, achieving excellent outcomes with minimal neurological deficits at discharge, and no specific safety concerns were identified. Complete symptom resolution was reported in two patients who received MT [27,28], one patient was discharged on foot [29], and one patient required short-term rehabilitation [30]. The remaining patients had minimal neurological deficits not affecting quality of life with NIHSS ≤ 2 [31,32,33,34,35,36,37,38]. Two patients received both tPA and MT: one had a significant deficit and died within three months [39], while the other fully recovered [37]. None of the case reports adopted a conservative management approach and the long-term antiplatelet regimen was not described in any of the included studies.

### 3.2. Observational Studies

Four observational studies with a total of 1598 patients were included and are summarized in Table 2.

Cline et al. compared tPA (*n* = 8) to conservative management (*n* = 14) [40]. Baseline characteristics were similar between both groups. No significant differences were observed in the median discharge NIHSS, mRS, and binary mRS variables (0–2 and >3). Additionally, the 90-day outcomes including new ischemic stroke, myocardial infarction, congestive heart failure, and death were similar between both groups. Higher rates of femoral bleeding were seen in the tPA group (75% vs. 0%, *p* = 0.005).

Levi et al. compared conservative management (*n* = 109) with neurointervention (tPA or MT, *n* = 36) in patients suffering moderate-to-severe AISCT [41]. Baseline NIHSS was significantly higher in the neurointervention group (14 (IQR 9–18)) compared to the conservatively managed group (4 (IQR 2–7), *p* < 0.001). No significant difference in one-year mortality was observed (58.9% vs. 52.4%, *p* = 0.78). Neurointervention was associated with a higher proportion of patients alive and independent at 90 days when compared to conservative management, showing a trend towards better survival and functional outcomes (36.1% vs. 22%, *p* = 0.12). In a logistic regression model controlling for stroke severity and Society of Thoracic Surgeons score, neurointervention was associated with an OR of 2.9 (95% CI: 1.2–7.0; *p* = 0.016) for independent survival at 90 days. The rates of major and life-threatening bleeding were similar. There was a trend towards higher rates of intracranial emorrhage in the neurointervention group, which did not reach statistical significance (8.6% vs. 2.2%, *p* = 0.08).

AlKhouli et al. compared conservative management (*n* = 1031), with tPA (*n* = 54) and MT (*n* = 50) [31]. Baseline NIHSS was not reported; however, the patients treated with MT/tPA were described as typically higher risk with higher stroke severity. In-hospital mortality was highest for MT (22%), followed by tPA (13%) and conservative management (7.7%) (*p* = 0.001). Cerebral hemorrhage was more frequent in the MT group (28%) compared to tPA (11.1%) and conservative management (3.2%) (*p* < 0.001). A higher proportion of conservatively managed patients were discharged home compared to tPA and MT (43.5% vs. 31.9% vs. 30.8%, respectively, no statistical test reported).

Khera et al. compared conservative treatment (*n* = 44), tPA (*n* = 3) and MT (*n* = 7) [42]. Baseline NIHSS scores were 14 (IQR 9–21) in the tPA or MT group, and 5 (IQR 3.5–9) in the conservative group. The patients treated with tPA or MT were typically younger, less frail, and had higher NIHSS when stroke-code was activated (*p* < 0.05). A significant reduction in NIHSS score was seen in both the tPA and MT groups (pre-treatment median NIHSS: 14, post-treatment median NIHSS: 5, *p* = 0.002). tPA was associated with more bleeding complications, requiring surgical intervention in two cases. There was no significant improvement in the NIHSS for the patients managed conservatively.

## 4. Discussion

Despite advances in procedural technique, valve and delivery system technology, and operator experience, stroke remains an infrequent but devastating complication of TAVI [7,43,44,45,46]. Limited progress has been made on stroke prevention in TAVI, with even less focus on treatment options. This systematic review evaluated the evidence base for the management of AISCT. We found a stark paucity of evidence to guide management decision making, with data limited to a small number of case reports, case series, and observational studies.

Given the clinical need for timely decision making in AISCT and a lack of high-quality evidence and guidelines, we constructed a working group of structural interventional cardiologists, stroke physicians, and neurointerventional radiologists to evaluate the evidence and provide practical guidelines on the management of AISCT.

### 4.1. Treatment Recommendations

There are four main treatment considerations in the hyperacute management of ischemic stroke: (1) acute stroke recognition, (2) conservative treatment (antiplatelet therapy and anticoagulation), (3) mechanical thrombectomy, and (4) thrombolytic therapy [20]. We will discuss each of these in turn in the context of the specific challenges in AISCT.

***(1)*** ***Acute stroke recognition and general management***

The included case reports highlight that AISCT can be identified at various points along the patient journey and by different members of the multidisciplinary team. The early identification and treatment of stroke is essential to improve outcomes [47]. Early stroke recognition should be simpler in the current era of TAVI given these procedures are no longer routinely performed under general anesthesia, and often not even under sedation. However, to identify acute stroke in a timely manner, all the clinical staff working with TAVI patients should have basic training in stroke recognition. We suggest a simple and validated screening tool, such as the “Balance, Eyes, Face, Arm, Speech and Time (BE-FAST) to act” public health initiative [48,49,50].

In assessing patients with a suspected stroke, clinicians should consider common stroke mimics, including hypoglycemia and seizure [21,51,52,53,54]. While a mimic may be less likely in the post-TAVI setting, simple tests routinely included in the assessment of acutely unwell patients, including capillary blood glucose, should not be overlooked [51,52,53,54]. Some patients presenting with a large stroke syndrome have a reduced GCS score and anesthetic support may be needed if the airway is compromised [51,54,55]. Swallowing issues are common during acute stroke and patients should be kept nil-by-mouth and enteral medications avoided until a swallow assessment has been carried out by a trained professional [51].

Stroke patients should be transferred to hyperacute stroke services as soon as possible. Cardiology teams must recognize the importance of timely intervention to rescue brain tissue and should be familiar with time “windows” for intervention. In the UK, this includes <9 h for tPA and <24 h for MT [51]. Local pathways and standard operating procedures should be established to help avoid delays in care, which will be dependent on the local arrangement of specialist services. In our institution, TAVI is performed at a separate hospital site to hyperacute stroke services, and therefore, immediate discussion with the HASU team and transfer to the HASU is required, with all neuroimaging typically taking place on arrival at the HASU site.

**Figure 2:**
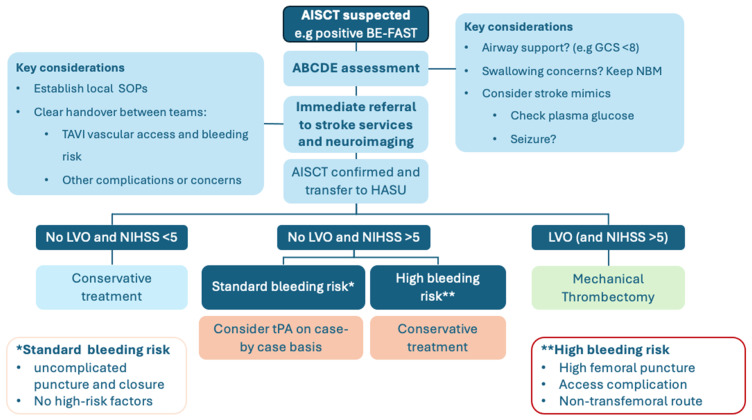
Summary of treatment recommendations for the management of AISCT.

Clear and efficient handover between specialties is essential. This includes the salient medical background (including a history of atrial fibrillation, anticoagulation history including the timing of the last dose, and any history of previous ischemic or hemorrhagic stroke), procedural details (such as primary and secondary access sites, antithrombotic medication and the timing of reversal agents used, and any specific concerns), and stroke details (including the timing of symptom onset/when was the patient last seen well, and a description of the stroke syndrome). A summary of our recommendations for stroke recognition and initial management is provided in Figure 2.

***(2)*** ***Conservative treatment***

In the United Kingdom, routine post-stroke antiplatelet therapy is with aspirin 300 mg once daily for 2 weeks, followed by clopidogrel (75 mg) lifelong [21,51]. Antiplatelets should be initiated as soon as possible once intracerebral hemorrhage has been excluded. In practice, if tPA is given, aspirin administration is often delayed until a CT head has been performed at 24 h to exclude hemorrhagic transformation. Aspirin is given rectally or via an enteral tube in patients with dysphagia. In patients with a minor stroke (and hence a lower risk of hemorrhagic transformation), dual antiplatelet therapy is sometimes considered [51].

Antiplatelet agents are also used routinely in TAVI to reduce ischemic events including stroke, MI, and valve thrombosis [56,57]. Aspirin monotherapy (300 mg loading pre-TAVI, followed by 75 mg once daily) is now the recommended antiplatelet strategy after TAVI (Class Ia), with DAPT only recommended if there has been recent coronary stenting [1,2,58], given no difference in ischemic events but less vascular complications with single antiplatelet therapy [58,59,60,61].

There is no randomized data to demonstrate the superiority of a particular antiplatelet strategy in the setting of AISCT. As demonstrated in large observational studies, most patients with acute stroke post-TAVI are treated conservatively [42]. Given current recommendations around routine antiplatelet therapy following both stroke and TAVI, usual stroke management should apply in large infarcts (aspirin monotherapy). The higher dose (300 mg) might be associated with some increased bleeding risk in post-TAVI patients, which should be monitored [1,2,51,56,57]. In small stroke syndromes, the neurological benefit of DAPT would need to be weighed against the small but significantly increased risk of TAVI bleeding complications [1,2,21,46,56,57]. Discussion between cardiology and stroke teams in this instance is recommended.

Anticoagulant (e.g., direct oral anticoagulant) monotherapy is the recommended strategy in patients undergoing TAVI with an indication for formal anticoagulation, such as atrial fibrillation [56,57]. The decision to hold this and switch to antiplatelet therapy in the acute post-stroke period (due to the risk of hemorrhagic transformation) will need cross-specialty discussion to balance the hemorrhagic and ischemic risk specific to the individual patient. In all TAVI patients, heparin is routinely given during the procedure to prevent catheter- and wire-associated thrombosis and embolization; however, this is usually at least partially reversed with protamine sulfate prior to vascular closure to reduce bleeding complications [59,60,61]. A summary of the recommendations for conservative management of stroke is provided in Figure 2.

***(3)*** ***Mechanical thrombectomy***

Mechanical thrombectomy can be performed in selected patients within 24 h of acute stroke symptom onset. It is the recommended treatment strategy for patients with large vessel occlusion who present early after the stroke before irreversible ischemic damage to the brain [21,51,62]. The strong evidence to support the use of MT as the standard of care in this context can be extrapolated to include the AISCT population. This requires local protocols and close multidisciplinary work, and should include timely transfer to hyperacute stroke services, early CT cerebral angiography, and discussion with the neurointerventional radiology team. To be eligible for MT, the current guidelines suggest patients should be previously independent in activities of daily living (mRS < 3). Given that aortic stenosis has a significant impact on functional status and frailty, with the expectation of an improvement in a large proportion of patients following TAVI, frailty cut-offs in the AISCT require careful consideration. A summary of our recommendations for MT following AISCT is provided in Figure 2.

***(4)*** ***Thrombolytic therapy***

In the general acute stroke population, tPA is recommended in significant stroke syndromes (NIHSS > 5), when hemorrhage has been excluded and the patient is within the 9 h window (with advanced imaging) from symptom onset [21,51]. Decisions around tPA in AISCT are more nuanced, and at present, there is no convincing evidence to support routine use given significant safety (bleeding) concerns. Major unanswered questions with regard to both safety and efficacy remain, and further data in this area are needed.

Bleeding with tPA therapy is a major concern in AISCT given the recent large bore (usually common femoral) arterial access. However, the bleeding risk will vary on a patient-by-patient basis (from moderate to prohibitive) and careful communication between specialties is essential. For example, if the femoral arterial puncture was high (above the level of the inguinal ligament), or a non-transfemoral primary access site was used, any bleeding may not be compressible and could be life-threatening. These risks need to be balanced against potential neurological benefits.

tPA has been shown to be effective in reducing stroke severity in the general population [63,64,65]. However, in addition to safety concerns, specific considerations are needed around the potential efficacy of tPA in the AISCT population [1,2]. The etiology of AISCT has not been fully established, but a significant proportion is thought to be caused by the embolization of calcific valve material, which may not respond to tPA [11,12,13,14,15,16]. Other potential etiologies unlikely to respond to tPA include gaseous emboli [66,67] and periprocedural hypoperfusion causing ischemia in watershed areas [68,69]. While a proportion of patients may have infarcts secondary to thrombus (either in situ or embolic), identifying such patients acutely is not always possible with the clinical diagnostic tools available.

In our assessment of the available literature, unlike with MT where in appropriately selected cases there is clear potential for benefit, there remains equipoise regarding the role of tPA for AISCT. We suggest that at present, tPA should be very cautiously considered on a patient-by-patient basis, and further data from an RCT in this area may be helpful. At present, given the lack of convincing evidence for the benefit of tPA in this setting and the clear potential for harm, tPA should only be considered when the risk of bleeding is assessed as moderate (i.e., simple routine closure with no high-risk features). A summary of our recommendations for tPA following AISCT is provided in Figure 2.

### 4.2. Post-TAVI Care on the HASU

**Figure 3:**
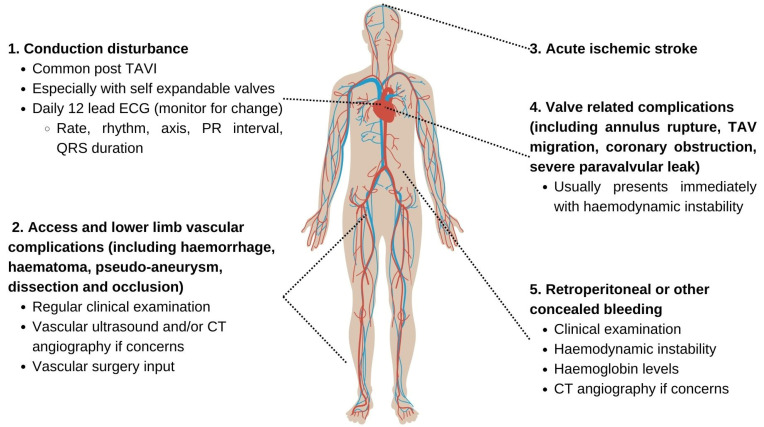
Summary of complications following TAVI. Common complications following TAVI include (1) conduction disturbance, (2) vascular complications related to large-bore arterial (usually transfemoral) access, and (3) acute ischemic stroke. Less common complications include complications related to the transcatheter aortic valve (TAV) itself (4), such as annulus rupture at the time of implantation (usually fatal), TAV migration, coronary obstruction, or severe paravalvular leak. These complications usually present immediately following valve deployment with hemodynamic instability and/or chest pain. Bleeding can sometimes be concealed (5), such as retroperitoneal hemorrhage, which can be seen following a high transfemoral puncture, and will be suspected by careful history (e.g., back pain), examination findings (e.g., Cullen’s or Grey Turner’s sign), and confirmed by computed tomography (CT) angiography.

All clinicians working in a HASU accepting post-TAVI patients require a working understanding of the routine management of patients post-TAVI and should be able to identify common complications that can occur, with the daily support of local cardiology teams. Common post-TAVI complications are summarized in Figure 3.

### 4.3. General Principles

There is a clear need for shared decision making and clear communication between teams and the patient or their relatives, given the lack of evidence and potential for harm. A similar multidisciplinary protocol was developed in the USA [42]. A streamlined decision-making tool led to a significant increase in the use of timely MT and tPA neurointerventions with a 35% absolute improvement in functional independence with MT (95% CI: 25% to 45%, *p* = 0.01) and a 25% improvement with tPA (95% CI: 15% to 35%, *p* = 0.03). These findings underscore the importance of improving AISCT management.

### 4.4. Limitations

The included studies were mostly case reports or small retrospective observational studies, with no randomized controlled trials. These data are, therefore, subject to the inherent biases associated with observational data, and causal links between treatment options and clinical outcomes cannot be established. Additionally, there is significant geographical variability in the availability of stroke services both internationally and within individual countries, which will impact the generalizability of these findings. There is likely a large body of unreported data on the use of tPA and MT following TAVI given that these are routine treatment options following acute ischemic stroke.

## 5. Conclusions

AISCT is an infrequent but devastating complication of TAVI. Prevention strategies have, to date, yielded disappointing results, and therefore, a focus on treatment is also needed. There is very limited evidence to guide management in AISCT. We established a working group to provide the first European multidisciplinary recommendations for the contemporary management of AISCT, outlining the specific considerations in AISCT for conservative management, tPA, and MT. Caution is needed, particularly when considering tPA, and further high-quality research may be helpful. Careful clinical consideration and close multidisciplinary work are needed to weigh up the specific risks and potential benefits of stroke interventions for the individual patient with AISCT.

## Figures and Tables

**Table 1:** Baseline patient characteristics and summary of findings from case reports and series.

Case Reports and Case Series	tPA (*n* = 4)	MT (*n* = 12)	tPA and MT (*n* = 2)
Age (mean +/− SD, 95% CI)	85.25 ± 4.262	81.4167 ± 5.035	78.5 ± 0.98
Sex (Male)	2	9	0
Sex (Female)	2	4	2
Baseline NIHSS	8.5 ± 3.279	18.3333 ± 2.836 **	21 (1 patient), Comatose (1 patient)
Baseline mRS *	-	-	-
Hypertension	2	0	0
Diabetes	0	0	0
Coronary heart disease	2	2	0
Smoking	0	0	-
Hypercholesterolemia/Dyslipidemia	1	0	0
Obesity (BMI > 30)	0	0	1
Known atrial fibrillation	1	0	0
Previous TIA	1	0	0
Previous stroke	0	0	0
TAVI device type -Self-expandable (n)	Not reported	5	-
TAVI device type - Balloon-expandable (n)	Not reported	5 (TAVI device type was not reported in 2 patients)	1 (TAVI device type was not reported in 1 patient)
Transfemoral access for TAVI	3 ^†^	7 ^†^	2
Cerebral embolic protection ^††^	-	-	-
**Stroke Recognition**			
Immediately post valve deployment	1	2	0
Immediately after valvuloplasty	1	2	0
<24 h after procedure	2	3	1
On reversal of general anesthesia	0	4	0
Less clear/not mentioned	0	2	1
**Stroke Management Outcomes**	Successful, NIHSS reduction, and no long-term complications	Excellent outcomes and minimal deficit	Mixed outcomes: 1 significant deficit and 1 full recovery
Safety outcomes	Significant bleeding from the femoral access site and bilateral groin hematomas were noted in the patients treated with tPA	No safety concerns were raised in the patients treated with MT	One patient died within three months

* none of the case reports or series comment on baseline functional or frailty status. ** only 6 studies included a baseline NIHSS. ^†^ the remainder of the studies did not mention the access site for TAVI. ^††^ none of the case reports or case series comment on the use of cerebral embolic protection devices.

**Table 2:** Summary of the main findings of the observational studies included.

Study	Patient Groups	Baseline NIHSS (Median)	Primary Outcomes	Bleeding/Complications	Other Comments
Cline et al. [40]	tPA (*n* = 8) vs. conservative (*n* = 14)	tPA: 3 (IQR 0–15) conservative: 5.5 (IQR 4–7) *	- No significant differences in adverse neurological or cardiac outcomes at discharge and 90 days. - Groin bleeding: tPA 75% vs. conservative 0% (*p* = 0.005) - Total bleeding events: tPA 88% vs. conservative 50% (*p* = 0.08) - Drop in hemoglobin ≥2 g/dL: tPA 75% vs. conservative 36% (*p* = 0.08)	- Symptomatic intracranial hemorrhage: tPA 0% vs. conservative 14% (*p* = 0.26) - New CHF: tPA 0% vs. conservative 14% (*p* = 0.26) - Groin bleeding: tPA 75% vs. conservative 0% (*p* = 0.005) - Drop in hemoglobin ≥2 g/dL: tPA 75% vs. conservative 36% (*p* = 0.08)	tPA group had more groin bleeding, but no long-term functional impairment. tPA appears safe for AIS post-TAVI, though more evidence is needed for efficacy.
Levi et al. [41]	Conservative (*n* = 109) vs. Neurointervention (*n* = 36)	Conservative: 4 (IQR 2–7) Neurointervention: 14 (IQR 9–18) **	- A trend towards better functional outcomes at 90 days in the neurointervention group, higher independence. In a logistic regression model controlling for stroke severity and Society of Thoracic Surgeons score, neurointervention was associated with an OR of 2.9 (95% CI: 1.2–7.0; *p* = 0.016) for independent survival at 90 days. - Independent (mRS 0–2) at 90 days: conservative 22% vs. Neurointervention 36% (*p* = 0.22)	- Vascular complications (minor): conservative 11.2% vs. Neurointervention 17.1% (*p* = 0.61) - Intracranial hemorrhage: conservative 2.2% vs. Neurointervention 8.6% (*p* = 0.08)	The neurointervention group has displayed a trend towards better functional outcomes at 90 days; however, more robust RCTs with larger patient groups are required to ascertain this finding.
AlKhouli et al. [31]	Conservative (*n* = 1031) vs. tPA (*n* = 54) vs. MT (*n* = 50)	Not reported	- Higher in-hospital mortality in the MT group. - In-hospital death: conservative 7.7% vs. tPA 13.0% vs. MT 22.0% (*p* = 0.001) - Cerebral hemorrhage: conservative 3.2% vs. tPA 11.1% vs. MT 28.0% (*p* < 0.001) - Blood transfusion: conservative 10.4% vs. tPA 20.4% vs. MT 16.0% (*p* = 0.04)	- Vascular complications: conservative 5.5% vs. tPA 13.0% vs. MT 6.0% (*p* = 0.08) - Permanent pacemaker: conservative 11.1% vs. tPA 11.1% vs. MT 10.01% (*p* = 0.97)	The higher severity of strokes in the tPA and MT groups likely contributed to differences. More patients in the conservative group were discharged home, with a significant selection bias noted.
Khera et al. [42]	tPA (*n* = 3) vs. MT (*n* = 7) vs. conservative (*n* = 44)	tPA or MT: 14 (IQR 9–21) conservative: 5 (IQR, 3.5–9)	- Significant reduction in NIHSS (pre-post): tPA or MT 14 (IQR 9–21) to 5 (IQR 1–8) (*p* = 0.002) conservative management has shown similar pre- and post- NIHSS	- Major bleeding requiring surgical intervention: tPA 2 vs. MT 0 - Major bleeding not requiring surgical intervention: tPA 1 vs. MT 0 - Hemorrhagic transformation: tPA 0 vs. MT 1	Significant reduction in NIHSS in neurointervention group was noted, with no significant difference noted in the conservative group. Higher risk of bleeding complications was noted with tPA.

* Cline et al.: baseline NIHSS were not significantly different between groups. ** Levi. et al.: baseline NIHSS was significantly higher in the neurointervention group.

## Abbreviations

NIHSS	National Institutes of Health Stroke Scale
mRS	Modified Rankin scale
TAVI	Transcatheter Aortic Valve Implantation
tPA	Tissue plasminogen activator
MT	Mechanical thrombectomy
